# Exomeres and supermeres: Monolithic or diverse?

**DOI:** 10.1002/jex2.45

**Published:** 2022-06-03

**Authors:** Juan Pablo Tosar, Alfonso Cayota, Kenneth Witwer

**Affiliations:** ^1^ Analytical Biochemistry Unit Nuclear Research Center School of Science Universidad de la República Montevideo Uruguay; ^2^ Functional Genomics Unit Institut Pasteur de Montevideo Montevideo Uruguay; ^3^ Department of Medicine University Hospital Universidad de la República Montevideo Uruguay; ^4^ Department of Molecular and Comparative Pathobiology Johns Hopkins University School of Medicine Baltimore Maryland USA; ^5^ Department of Neurology Johns Hopkins University School of Medicine Baltimore Maryland USA

**Keywords:** extracellular nanoparticles, extracellular ribosomes, non‐vesicular RNA

## Abstract

Extracellular vesicles (EVs), including exosomes and microvesicles, are far from being the only RNA‐containing extracellular particles (EPs). Recently, new 35‐nm‐sized EPs were discovered by asymmetric‐flow field‐flow fractionation and termed ‘exomeres’. Purification of exomeres was later performed by differential ultracentrifugation as well. More recently, the supernatant of the high‐speed ultracentrifugation used to collect exomeres was further centrifuged to collect a new class of EP, termed ‘supermeres’. Supermeres contain high quantities of extracellular RNA and are enriched in miR‐1246. They are also replete in disease biomarkers and can induce metabolic and adaptive changes in recipient cells. Here, we reanalysed proteomic and transcriptomic data obtained in this exciting study to obtain further insights into the molecular composition of exomeres and supermeres. We found that the top‐ranking RNAs in supermeres correspond to the footprints of extracellular protein complexes. These complexes protect fragments of the small nuclear RNA U2 and the 28S rRNA from extracellular ribonucleases (exRNases). We suggest that intracellular nanoparticles such as the U2 ribonucleoprotein, ribosomes and LGALS3BP ring‐like decamers are released into the extracellular space. These heterogeneous EPs might be further processed by exRNases and co‐isolate by ultracentrifugation with other components of exomeres and supermeres. We look forward to continuing progress in defining exRNA carriers, bridging process definitions with molecular composition and function.

## 1 INTRODUCTION

Interest in extracellular RNA (exRNA) has ramped up in the last 15 years due to their involvement in intercellular communication and promising use in diagnostics (Li et al., 2018; Mateescu et al., 2017). Extracellular vesicles (EVs) are nanosized cell‐derived biological particles defined by a lipid bilayer and capable of transferring exRNAs from one cell to another (O'Brien et al., 2020). For years, EVs have been the most widely studied exRNA carriers in the extracellular space, despite early recognition of exRNA transport in the context of lipoprotein particles (Vickers et al., 2011) and lipid‐free ribonucleoproteins (RNPs) (Arroyo et al., 2011; Turchinovich et al., 2011). However, it was not until the recent discovery of ‘exomeres’ (Zhang et al., 2018, 2019) that nonvesicular extracellular particles (EPs) gained momentum (Hoshino et al., 2020).

Exomeres were first separated by asymmetric‐flow field‐flow fractionation (AF4) as a new class of non‐membranous EP approximately 35 nm in diameter (Zhang et al., 2018). Exomeres were found to contain a characteristic set of proteins, including glycolytic enzymes and proteins involved in glycan processing and recognition. In an independent effort, Robert Coffey's group optimised an ultracentrifugation‐based method to purify exomeres without the need for AF4 and studied their capacity to transfer functional cargo between cells (Zhang et al., 2019). In a recent and very exciting article published in *Nature Cell Biology*, these authors went a step forward and subjected the supernatant of the exomere preparation to ultracentrifugation at higher speeds and collected a new class of EP: the supermeres (‘supernatant of exomeres’) (Zhang et al., 2021). The authors showed that the protein and RNA composition of supermeres is distinct to that of exomeres and EVs, and contain multiple cargo involved in several chronic diseases. Supermeres also harbour the majority of the exRNA in the conditioned media of colorectal cancer DiFi cells, can cross the blood–brain barrier and can induce metabolic and adaptive changes in recipient cells, including the transfer of drug resistance.

Zhang et al. (2021) also observed a strong enrichment in supermeres of an RNA known as miR‐1246. This short RNA typically shows substantial 5′ heterogeneity, which is inconsistent with canonical miRNA biogenesis (Fromm et al., 2015). Indeed, as analysed in detail by the authors (Zhang et al., 2021), reads assigned to miR‐1246 actually correspond to the RNU2‐1 locus. That is, miR‐1246 is a fragment of the U2 small nuclear RNA (snRNA) (Tosar et al., 2017; Xu et al., 2019).

By analysing structural data from the spliceosome, here we show that miR‐1246 is the footprint of the Sm heptamer protecting an internal region of the U2 snRNA (RNU2). Consistent with this, silencing of splicing factors has been shown to affect extracellular miR‐1246 levels and U2 processing patterns (Xu et al., 2019). The fact that a fraction of the U2 RNP (probably containing fragmented RNA) co‐isolates with supermeres is illustrative of the intrinsic heterogeneity of these new extracellular nanoparticles. Considering antibodies against Sm proteins are frequent in autoimmune diseases (McClain et al., 2002), this analysis further connects supermeres with immune surveillance of potential cell‐death‐derived ribonucleoprotein complexes. We also provide evidence supporting the presence of other discrete nanoparticles in exomeres and supermeres, including ribosomes or ribosomal subunits and ring‐like LGALS3BP decameric structures.

## 2 RESULTS

### 2.1 The extracellular U2 RNP is likely the source of miR‐1246 in supermeres

**FIGURE 1:**
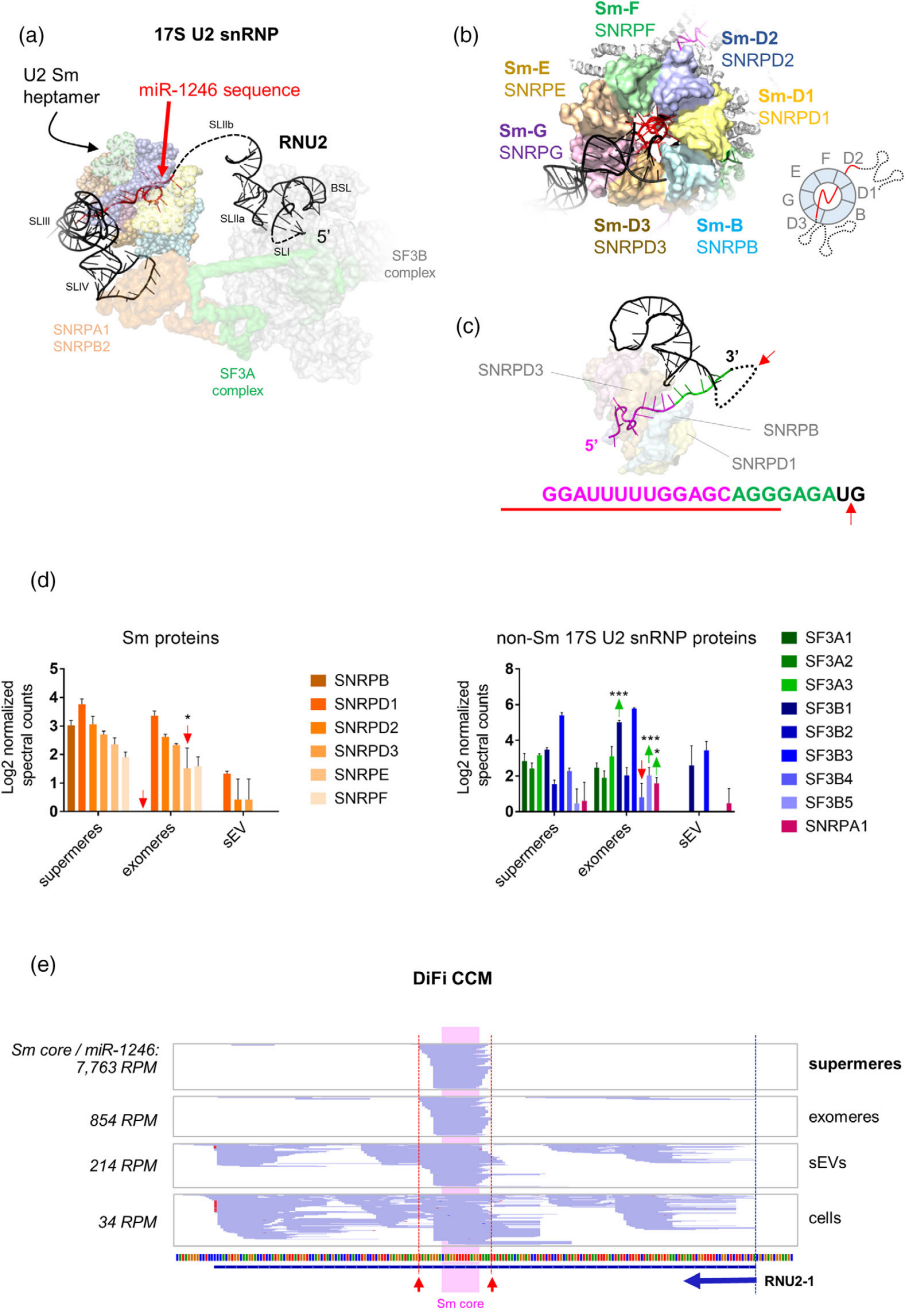
Extracellular miR‐1246 corresponds to the footprint of the Sm heptamer protecting the oligo(U) sequence motif of the U2 small nuclear RNA. (a) Cryo‐EM structure of the 17S U2 snRNP (PDB: 6Y5Q) showing the sequence of miR‐1246 in red. (b) Three‐dimensional model of the Sm heptamer, forming a ring around the miR‐1246‐like sequence of RNU2 (based on the structure of the precursor pre‐catalytic spliceosome, PDB: 6AH0). (c) The sequence of RNU2 protected by the Sm ring is shown in magenta. Purines extending toward the 3′ end are shown in green. The red arrow represents the putative cleavage site by members of the RNase A superfamily. The red line represents the sequence of the mature hsa‐miR‐1246 according to miRBase. (d) Normalised spectral counts of Sm proteins (left) and U2‐associated splicing factors (right) in supermeres, exomeres and EVs from DiFi cells, based on data provided in Zhang et al. (2021). Proteins showing statistically higher (green arrows) or lower (red arrows) normalised spectral counts in exomeres versus supermeres are indicated (two‐way ANOVA with Tukey's multiple comparison test). ^*^ *p* < 0.05. ^***^ *p* < 0.001. (e) Coverage plots of RNU2‐1 in supermeres, exomeres, EVs and DiFi cells (replicate 1), based on small RNA sequencing data provided in Zhang et al. (2021). The blue arrow indicates the start and direction of RNU2‐1. Note the blue‐coloured reads correspond to the minus DNA strand, and their orientation (5′‐3′) is therefore right‐to‐left. Red arrows indicate predictive cleavage sites (by RNase A family members) 3′ to the most proximal pyrimidines outside of the Sm core region.

We reasoned that better understanding of the association between miR‐1246 and supermeres would shed light on the composition of these newly described EPs. Strikingly, analysis of cryo‐EM structures of the human 17S U2 snRNP (Zhang et al., 2020) and the precursor pre‐catalytic spliceosome (Zhan et al., 2018) show that miR‐1246 corresponds to the footprint of the heptamer of Sm proteins on RNU2 (Figure 1a–c and Supplementary Figure S1a). Sm proteins form a ring‐like structure around oligo(U) sequences present in several snRNAs, including RNU2 and RNU4 (Achsel et al., 2001). However, the stretch of single‐stranded bases expanding beyond the Sm site before the next pyrimidine is longer in RNU2, explaining the observed bias towards U2 snRNA‐derived fragments (Figure 1c and Supplementary Figure S1b). Considering that members of the ribonuclease A superfamily are abundant in extracellular samples (Lu et al., 2018) and cut on the 3′ side of pyrimidine residues (Raines, 1998), we hypothesised that extracellular miR‐1246 is the result of extracellular cleavage of the U2 snRNA in complex with a ring‐shaped Sm heptamer.

Consistent with our hypothesis, we found six out of seven core Sm proteins in the reported proteome of supermeres (Figure 1d). Normalised spectral counts correlated with protein molecular weight, with SmG (SNRPG) being the only undetected protein (and the smallest). Sequence coverage of the longest Sm protein (SNRPB) was restricted to the N‐terminal half of the protein. Although this could be indicative of partial proteolysis, it can also be a consequence of inefficient trypsin digestion deriving from the lack of C‐terminal Lys residues combined with extensive arginine methylation in this region (Li et al., 2021), which is required for snRNP assembly (Brahms et al., 2001).

Other constituents of the 17S U2 snRNP were also present in supermeres and exomeres, including members of splicing factors 3a (SF3A) and 3b (SF3B). Interestingly, SF3B1 and SNRPA1 (small nuclear ribonucleoprotein A′) were enriched in exomeres compared with supermeres. Considering that SF3B1 is the largest subunit of SF3B and interacts with the branch site loop (BSL) at the 5′ half of RNU2 (Supplementary Figure S1c), loss of this protein could explain the lower observed sedimentation coefficient of supermeres compared with exomeres. This model also predicts that fragments arising from the BSL (i.e. SF3B1‐protected fragments) and the stem loop IV of RNU2 (i.e. SNRPA1‐protected fragments) should be observed in exomeres rather than supermeres. Indeed, coverage plots of RNU2 based on small RNA sequencing data in Zhang et al. (2021) supported this prediction (Figure 1e). While supermeres contained only the Sm‐protected sequence (with putative cleavage sites at the 3′‐side of the most proximal pyrimidines), exomeres contained additional reads at the BSL and SL IV. In contrast, EVs showed a more homogeneous distribution of reads along the entire RNU2 transcript, consistent with the inaccessibility of extracellular ribonucleases (exRNases) to EV‐protected RNAs (Tosar et al., 2020).

Survivorship bias defines the non‐vesicular extracellular RNAome (Tosar et al., 2021a). This observation was further affirmed when we compared RNU2 coverage plots in extracellular nonvesicular samples obtained from wild‐type or RNAse1‐null K562 cells (Nechooshtan et al., 2020), with an even distribution of reads in the absence of extracellular RNases (Supplementary Figure S2a). Analysis by RI‐SEC‐seq, a technique that preserves and separates extracellular RNPs according to their size under native conditions (Tosar et al., 2020), showed enrichment of Sm‐protected fragments in the ‘P0’ peak, corresponding to high molecular weight extracellular complexes (Supplementary Figure S2b).

To be sure, miR‐1246 is abundant in the supernatant of vesicle‐depleted foetal bovine serum (FBS) (Wei et al., 2016) and therefore, frequently appears as an extracellularly enriched miRNA in FBS‐containing samples (Tosar et al., 2017; Wei et al., 2016). However, miR‐1246 was also a major constituent of supermeres purified from the medium of cells grown under serum‐free conditions (Zhang et al., 2021), downplaying the likelihood of contamination as the sole explanation for this sequence. In supernatants from FBS, coverage plots of RNU2‐1 were virtually identical to those found in supermeres and were not perfectly aligned to the mature miR‐1246 sequence, as annotated in miRBase (Supplementary Figure S3a). In human serum, basal levels of miR‐1246/RNU2 are low in healthy individuals but are significantly enriched under pathological situations such as type 2 diabetes (Supplementary Figure S3b). Sequence coverage plots in the serum of these patients were comparable to those of supermeres and exomeres, with most reads corresponding to Sm‐protected fragments with putative cleavage sites 3′ to proximal pyrimidines (Supplementary Figure S3c). The presence of the U2 snRNP in the human circulation (indicated by the accumulation of Sm‐protected fragments) is also consistent with our recent identification of full‐length snoRNAs in the bloodstream (Tosar et al., 2021b).

### 2.2 Extracellular ribosomes co‐isolate with exomeres under serum‐free conditions

**FIGURE 2:**
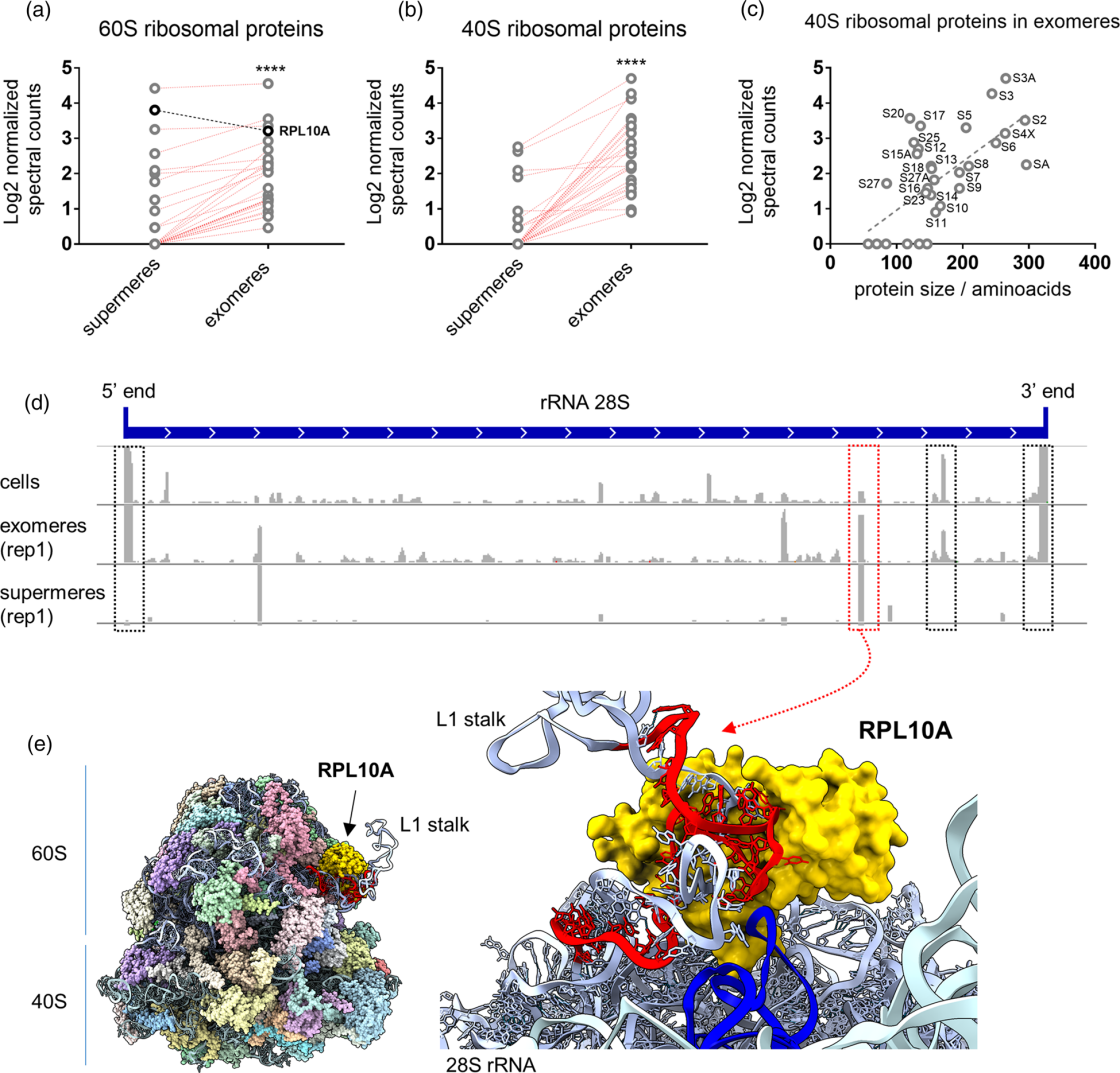
Analysis of ribosomal proteins and rRNA‐derived fragments in supermeres and exomeres. (a,b) Comparison of Log2‐transformed normalised spectral counts of 60S (a) and 40S (b) ribosomal proteins in supermeres and exomeres. Values correspond to the average of three replicates. Red lines: proteins producing a larger number of normalised spectral counts in exomeres than in supermeres. As a group, ribosomal proteins were more abundant in exomeres than in supermeres (Wilcoxon matched‐pairs signed rank test). ^****^ *p* < 0.0001. (c) Correlation between normalised spectral counts for 40S ribosomal proteins and protein size in amino acids. (d) Sequence coverage plots for the entire 28S rRNA (in 5′‐3′ orientation) in cells, exomeres and supermeres (replicate 1 for each). Black boxes: regions showing high coverage in cells and exomeres, but not in supermeres. Red box: region producing a rRNA‐derived fragment highly enriched in supermeres. (e) Cryo‐EM structure of the human ribosome (PDB: 4UG0), showing RPL10A in gold and the 28S rRNA‐derived fragment that is highly enriched in supermeres (red box in d) in red. Inset: close‐up view of the interaction between this sequence and RPL10A. All other ribosomal proteins were hidden. Blue: tRNA placed on the ribosomal E‐site

We have recently identified extracellular ribosomes (Tosar et al., 2020). Because extracellular ribosomes were collected in the same chromatographic peak where we also found the RNU2‐processing pattern that is characteristic of exomeres and supermeres (Supplementary Figure S2b), we asked whether these EPs could also contain ribosomes or ribosomal subunits. Indeed, mass spectrometry data collected by Zhang et al. (2021) from exomeres and supermeres included 26 proteins from the large (60S) and 25 proteins from the small (40S) ribosomal subunit, accounting for 55% and 78% of the total number of ribosomal proteins, respectively (Figure 2a,b). There was a significant correlation between protein size and normalised spectral counts for proteins of the small ribosomal subunit (Spearman's *r* = 0.5, *p* = 0.0032; Figure 2c). The correlation was weaker for 60S proteins, but still significant (Spearman's *r* = 0.35, *p* = 0.016). Thus, missing ribosomal proteins could still be present in the samples but might be too short and produce too few peptides for reliable detection. Normalised spectral counts for ribosomal proteins were always higher in exomeres compared to supermeres, except for RPL10A (Figure 2a,b).

Sequencing data were consistent with higher levels of full‐length rRNAs in exomeres compared to supermeres. For instance, the normalised number of reads mapping to the 5.8S rRNA was between 5‐ and 14‐fold higher in exomeres compared to the paired biological replicate of supermeres (Supplementary Figure S4a,b). Although the opposite trend was found for reads mapping to the 28S rRNA, sequence coverage plots of the 28S rRNA in exomeres were highly reminiscent to those inside cells (Figure 2d), with the highest sequence coverage accumulating at the 5′ and 3′ ends. In contrast, the fragmentation profile of supermeres was characterised by a sharp accumulation of reads mapping to a few discrete internal positions in the 28S rRNA.

It should be considered that small RNA‐seq is not capable of retrieving full‐length ribosomal RNAs. This does not mean that full‐length rRNAs are not present, and they may even be at great excess compared with sequenced fragments. To illustrate this point, when we used a read length of 200 nt (rather than the 50 nt reads used in Zhang et al., 2021) in samples containing extracellular ribosomes, we obtained coverage plots for the 28S rRNA similar to those shown in Figure 2d, but we also obtained several reads corresponding to the full‐length (157 nt) 5.8S rRNA (Tosar et al., 2020). The fact that the fragmentation profile of the 28S rRNA was very similar between exomeres and cells (where we assume that most of the rRNA is not fragmented) strongly suggests that exomeres contain full‐length rRNAs. In contrast, supermeres are enriched in some discrete and very specific rRNA fragments.

We then mapped the 28S rRNA internal sequence that is most enriched in supermeres on a cryo‐EM structure of the human ribosome and found it corresponds to a dsRNA region in the L1 stalk (Figure 2e). Furthermore, this protected rRNA fragment is accommodated inside a cleft in RPL10A, the only ribosomal protein enriched in supermeres compared to exomeres (Figure 2a). Although this is only correlative evidence, our interpretation is that extracellular ribosomes or ribosomal subunits are pelleted at the speeds used to collect exomeres, while fragmented ribosomal subunits containing rRNA fragments and their associated proteins remain in the supernatant and are pelleted as supermeres. This is analogous to our earlier reasoning regarding the U2 RNP.

If exomeres contain extracellular ribosomes, why were ribosomal RNAs not detected in the publication that first described exomeres (Zhang et al., 2018)? Beyond the use of different separation techniques (AF4 vs. dUC), the Lyden group's work was performed with cells incubated with vesicle‐depleted FBS (Zhang et al., 2018), while the Coffey lab purified exomeres and supermeres under serum‐free conditions (Zhang et al., 2021). We have shown that extracellular ribosomes are fragile entities and highly sensitive to the presence of serum‐derived ribonucleases in the media (Tosar et al., 2020), which may well explain this discrepancy. Fragmentation of ribosomes by exRNases would also impact the sedimentation coefficient of ribosomal proteins.

### 2.3 LGALS3BP is a marker of exomeres and a protein that self‐assembles into exomere‐sized particles

Galectin‐3‐binding protein (LGALS3BP, also known as Mac‐2 binding protein) was originally described as highly enriched in exomeres from a variety of human and murine cell lines (Zhang et al., 2018). More recently, Zhang et al. (2021) presented a heatmap of the most abundant proteins in small EVs, the whole nonvesicular fraction, exomeres and supermeres from DiFi cells (see Figure 2c in the reference publication). Close inspection of the data shows LGALS3BP among the few proteins specifically enriched in exomeres. Taken together, these results position LGALS3BP as a potential universal marker of exomeres. Furthermore, it has been speculated that this protein could mediate the specific interaction of exomeres with target cells (Zhang et al., 2018).

However, LGALS3BP is a protein of the extracellular matrix that self‐assembles into ring‐like decamers of 30–40 nm (Sasaki et al., 1998), consistent with the size of exomeres as determined by atomic force microscopy (Zhang et al., 2018, 2021). Similarly, the 97‐kDa VCP, also enriched in exomeres from DiFi cells, self‐assembles into hexamers with the shape of a flattened hourglass and a diameter of 15.6 nm (DeLaBarre & Brunger, 2003; Wang et al., 2003).

The case of LGALS3BP adds up to what we have previously shown for Sm and ribosomal proteins. It is remarkable that proteins enriched in exomeres are known to ensemble into particles that are roughly the same size as exomeres (∼35 nm). This raises the interesting question of whether exomeres and supermeres are homogeneous extracellular nanoparticles containing these proteins or a heterogeneous collection of compositionally distinct extracellular nanoparticles that tend to co‐isolate based on size.

## 3 DISCUSSION

The molecular, physicochemical and functional heterogeneity of EPs has been a matter of extensive research in the past 50 years. For instance, the four major classes of circulating lipoproteins (chylomicrons, VLDL, LDL and HDL) can be further divided into several biologically relevant subclasses (Kaess et al., 2008). The EV research field is younger, but remarkable progress has been made recently in understanding the functional implications of EV heterogeneity. An eruption of new technologies achieving single‐vesicle analysis (Bordanaba‐Florit et al., 2021) coupled to systematic proteomic characterisation of different vesicular subpopulations (Jeppesen et al., 2019; Kowal et al., 2016; Mathieu et al., 2021) are opening new horizons and securing a bright future for EV research. In addition, new separation techniques are being developed to increase the yield, throughput and purity of EV preparations. Nevertheless, differential ultracentrifugation has been the gold standard and remains the most popular method to isolate EVs (Royo et al., 2020), although it is widely accepted that vesicular populations collected by centrifugation are heterogeneous in terms of their biogenesis, size, composition and function (Thery et al., 2018). Unsurprisingly, process definitions of new particle classes are currently driving new discoveries of smaller‐than‐EV EP classes: exomeres (Zhang et al., 2018) and more recently supermeres (Zhang et al., 2021).

Our analysis of the proteomic and transcriptomic data obtained by Zhang et al. (2021) strongly suggests that exomeres and supermeres are a heterogeneous group of extracellular complexes that share similar sedimentation coefficients. Rather than a single nanoparticle replete with disease biomarkers, our interpretation is that components of the spliceosome, ribosomes and other abundant cellular complexes form a continuous spectrum of particles that co‐isolate with exomeres and supermeres or directly define subclasses.

Whether these complexes are actively released by live cells or passively released from dead cells remains to be determined, but the presence of nuclear proteins imposes mechanistic constrains to the former hypothesis. Nevertheless, differential ultracentrifugation has been the historical approach to identify new extracellular functional entities, and the available functional data on exomeres (Zhang et al., 2019) and supermeres (Zhang et al., 2021) illustrate the importance of considering intercellular communication mediated by EPs beyond the widely studied EVs.

## 4 METHODS

### 4.1 Small RNA sequencing data analysis

For most analysis on supermeres and exomeres, source data were retrieved from Zhang et al. (2021). When specifically indicated, unprocessed small RNA‐seq datasets were also retrieved from other studies (Krauskopf et al., 2017; Nechooshtan et al., 2020; Tosar et al., 2020; Wei et al., 2016). In all cases, selected Fastq files were downloaded from NCBI based on the accession numbers provided in each study (GEO: GSE168418 for supermeres, exomeres, sEVs and DiFi cells; GEO: GSE148516 for nonvesicular exRNAs in wt and RNase1‐null K562 cells; BioProject: PRJNA633249 for chromatographic fractions from MCF‐7 cell‐conditioned media containing or not RNase inhibitors; SRA: SRR3209615 for FBS ultracentrifugation supernatants and SRA: SRR5034619 for serum of a patient with type 2 diabetes mellitus).

Using FastQC (https://github.com/s‐andrews/FastQC), study‐specific 3′ adapter sequences were manually identified and removed using Cutadapt (https://github.com/marcelm/cutadapt). Adapter‐trimmed reads were then aligned to the relevant genome (human hg38 or cow bosTau8) using Bowtie2 (https://github.com/BenLangmead/bowtie2). Sequence coverage plots were visualised using the Java desktop application of the Integrated Genome Viewer (https://software.broadinstitute.org/software/igv/). The number of reads mapping to each ribosomal RNA was calculated using FeatureCounts (https://bio.tools/featurecounts) based on a gene annotation file downloaded from NCBI RefSeq via the UCSC table browser (https://genome.ucsc.edu/cgi‐bin/hgTables). MicroRNA relative abundances (expressed as million mapped reads, RPM) in the sera of patients with Hepatitis B virus (HBV) and type 2 diabetes mellitus (T2DM) were based on pre‐computed calculations included in liqDB (https://bioinfo5.ugr.es/liqdb).

### 4.2 Proteomic data analysis

Pre‐processed proteomic data containing spectral counts for each detected protein (normalised to total spectral counts and log2‐transformed) were downloaded from the supplementary materials section of Zhang et al. (2021). Sequence coverage and spectra supporting detection of Sm proteins was verified by analysing deposited raw data (project PXD025213 at https://www.ebi.ac.uk/pride/) using the software PatternLab (http://www.patternlabforproteomics.org/) and a human reference proteome downloaded from Uniprot. Statistical analysis was performed with GraphPad Prism v7.

### 4.3 Structural data analysis

CryoEM structures of human RNPs were downloaded from the Protein Data bank (https://www.rcsb.org/). Accession numbers were: 6Y5Q for the 17S U2 snRNP; 6AH0 for the precursor pre‐catalytic spliceosome; 4UG0 for the 80S human ribosome. Structures were analysed/visualised with ChimeraX (https://www.rbvi.ucsf.edu/chimerax/).

## Supporting information


**Supplementary Figure 1**: A) Cryo‐EM structure of the human precursor pre‐catalytic spliceosome (PDB: 6AH0). The sequence corresponding to miR‐1246 is shown in red. Most spliceosomal proteins and RNAs were hidden, except those corresponding to the U2 RNP, pre‐mRNA (magenta), RNU4 (orange), RNU6 (blue), and their associated Sm or LSm proteins, respectively. Protein surfaces were set to 80% transparency. BPS: branch point site. B) Close‐up view of the interaction between Sm proteins and RNU4. The sequence protected by the Sm ring is shown in magenta. Green bases correspond to additional 3′ purines extending out of the Sm ring until encountering the next pyrimidine residue (putative cleavage site by RNase A‐family members). C) Rotated view of (A), highlighting the interaction between SF3B1 (dark gray) and the branch site loop (BSL) of RNU2.

**Supplementary Figure 2**: A) Sequence coverage plot of RNU2‐1 in nonvesicular extracellular samples (100,000 x g supernatants) of RNase 1‐null (top) and wild‐type (bottom) K562 cells grown under serum‐free conditions. Source data is from Nechooshtan et al. (2020) (SRA: SRR11539127 and SRR11539120, respectively). The diagram represents Sm proteins and the U2 snRNA (red), and the action of extracellular ribonucleases (yellow). B) Sequence coverage plot of RNU2‐1 in nonvesicular extracellular samples from MCF‐7 cells, further separated by size‐exclusion chromatography. Chromatograms are shown on the left. Serum‐free, vesicle‐depleted cell‐conditioned medium (CCM) was either treated or not with ribonuclease inhibitors (+/‐ RI). Selected peaks were then subjected to small RNA‐seq: “P0” (corresponding to the exclusion volume of a Superdex 200 column) and “P1”, corresponding to the elution volume of free tRNAs and other RNAs or RNA fragments of similar size. Source data is from Tosar et al. (2020). As in Figure 1, the blue‐coloured reads correspond to the minus DNA strand and their orientation (5′‐3′) is therefore right‐to‐left. The blue arrow indicates the start and direction of RNU2‐1 and the Sm core sequence is indicated in magenta.

**Supplementary Figure 3**: A) Sequence coverage plot of two loci in the bovine genome corresponding to RNU2 or a RNU2 pseudogene (top) and miR‐1246 (bottom). The blue arrow indicates the start and direction of the pre‐miR‐1246 sequence. Reads in the forward orientation (red) are from SRRR3209615 (100,000 x g supernatants of 10% FBS; Wei et al. 2016). Mature miR‐1246 was shown in Wei et al. 2016 as the second most abundant miRNAs in FBS. However, reads mapping to miR‐1246 are derived from at least two genomic loci (based on the presence of a mismatch, indicated by an asterisk) and do not correspond to the mature bta‐miR‐1246 sequence (red bar). The Sm core sequence is indicated in magenta and predicted cleavage sites 3′ to the most proximal pyrimidines outside of the Sm core are indicated with an arrow. B) LiqDB analysis of hsa‐miR‐1246 (left), hsa‐miR‐122 (centre) and hsa‐miR‐486 (right) in the sera of healthy donors or patients with Hepatitis B virus (HBV) or type 2 diabetes mellitus (T2DM). RPM: reads per million mapped reads. Source data is from Krauskopf et al. (2017). Hsa‐miR‐122 is included as a control because it is known to be affected in HBV‐infected patients. Hsa‐miR‐486 is a red blood cell‐derived miRNA that is abundant in serum and serves to show the overall number of miRNA‐mapping reads in the three categories are comparable. Thus, T2DM patients seem to have specifically high levels of hsa‐miR‐1246. However, liqDB is counting reads derived from the RNU2‐1 locus as miR‐1246. When analysing sequence coverage plots (C) of the RNU2‐1 and the miR‐1246 loci in a T2DM patients with unusually high levels of miR‐1246 (SRA: SRR5034619), most of the reads correspond to RNU2‐1. Again, most of these reads correspond to the Sm core region (magenta) flanked by additional, mostly purine residues (predicted cleavage sites 3′ to proximal pyrimidines are indicated). However, some reads corresponding to the branch site loop (BSL) and the stem loop IIa (SLIIa) are also present, as well as some reads derived from the 3′ end of the RNU2‐1. As in Figure 1, the blue‐coloured reads correspond to the minus DNA strand and their orientation (5′‐3′) is therefore right‐to‐left. The blue arrow indicates the start and direction of RNU2‐1 (top) or pre‐miR‐1246 (bottom). The red bar indicates the sequence of the mature hsa‐miR‐1246 as annotated in miRbase.

**Supplementary Figure 4**: A) Sequence coverage plots of the 45S pre‐ribosomal RNA, containing the regions corresponding to the 18S, 5.8S and 28S rRNAs (from left to right in 5′‐3′ orientation). Source data is from Zhang et al. (2021). Unlike Figure 2D, the coverage plots were truncated at 300 reads (except in cells, where this number was set to 4000) in order to observe all the regions producing a relevant number of reads rather than those regions with the highest sequence coverage. B) Comparison of the total number of reads mapping to the 5.8 rRNA gene (in Log10 scale) in supermeres vs exomeres. Dashed lines connect paired samples (i.e., replicates 1, 2 and 3).
