# Supplementary figures and images for: Exomeres and supermeres: Monolithic or diverse?
Source: J Extracell Biol. 2022 Jun 3;1(6):e45. doi: 10.1002/jex2.45 (PMC9610496; doi:10.1002/jex2.45)

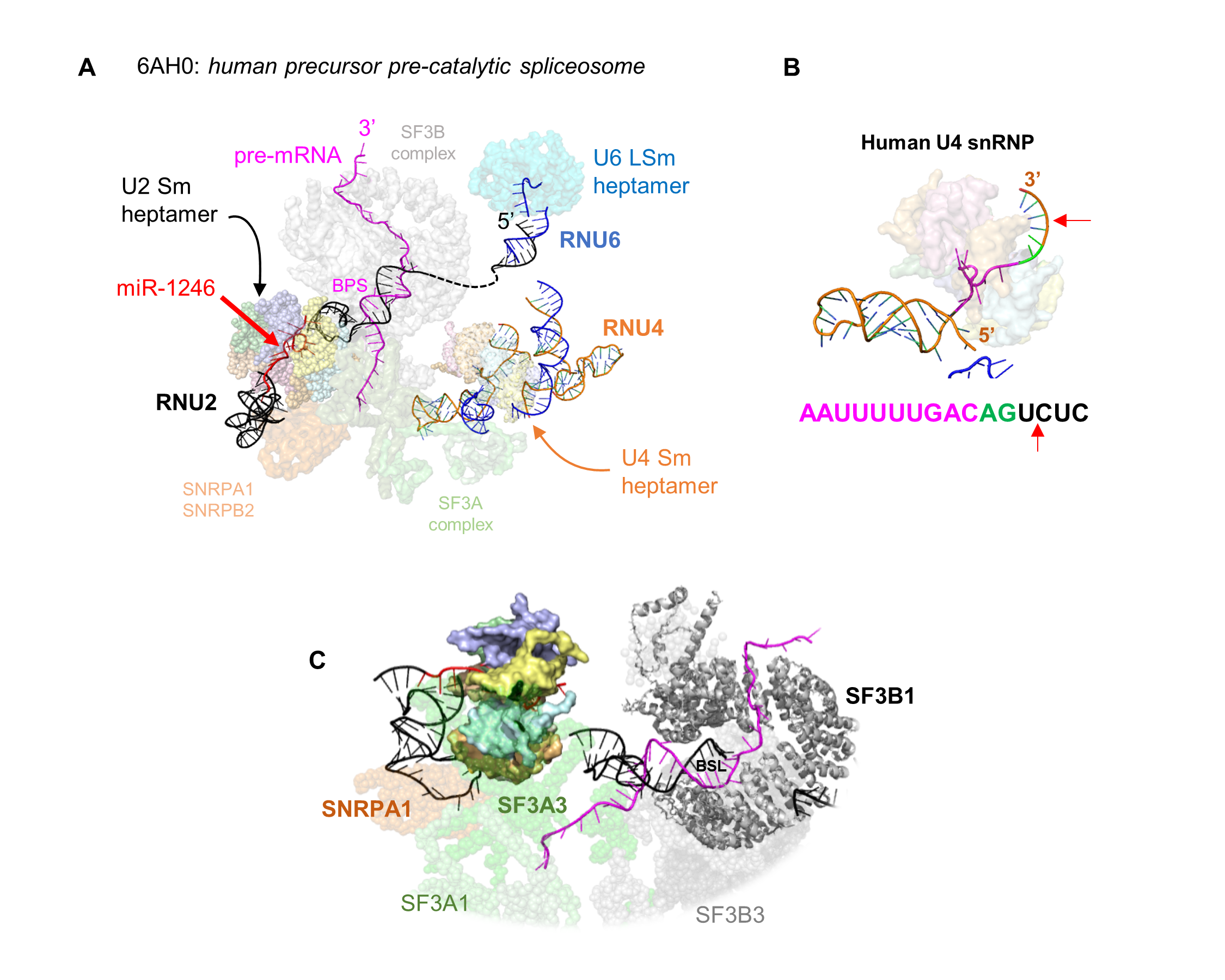

Supplement: Supplementary file 1 — Supplementary Figure 1: A) Cryo‐EM structure of the human precursor pre‐catalytic spliceosome (PDB: 6AH0). The sequence corresponding to miR‐1246 is shown in red. Most spliceosomal proteins and RNAs were hidden, except those corresponding to the U2 RNP, pre‐mRNA (magenta), RNU4 (orange), RNU6 (blue), and their associated Sm or LSm proteins, respectively. Protein surfaces were set to 80% transparency. BPS: branch point site. B) Close‐up view of the interaction between Sm proteins and RNU4. The sequence protected by the Sm ring is shown in magenta. Green bases correspond to additional 3′ purines extending out of the Sm ring until encountering the next pyrimidine residue (putative cleavage site by RNase A‐family members). C) Rotated view of (A), highlighting the interaction between SF3B1 (dark gray) and the branch site loop (BSL) of RNU2. [file JEX2-1-e45-s003.tif]

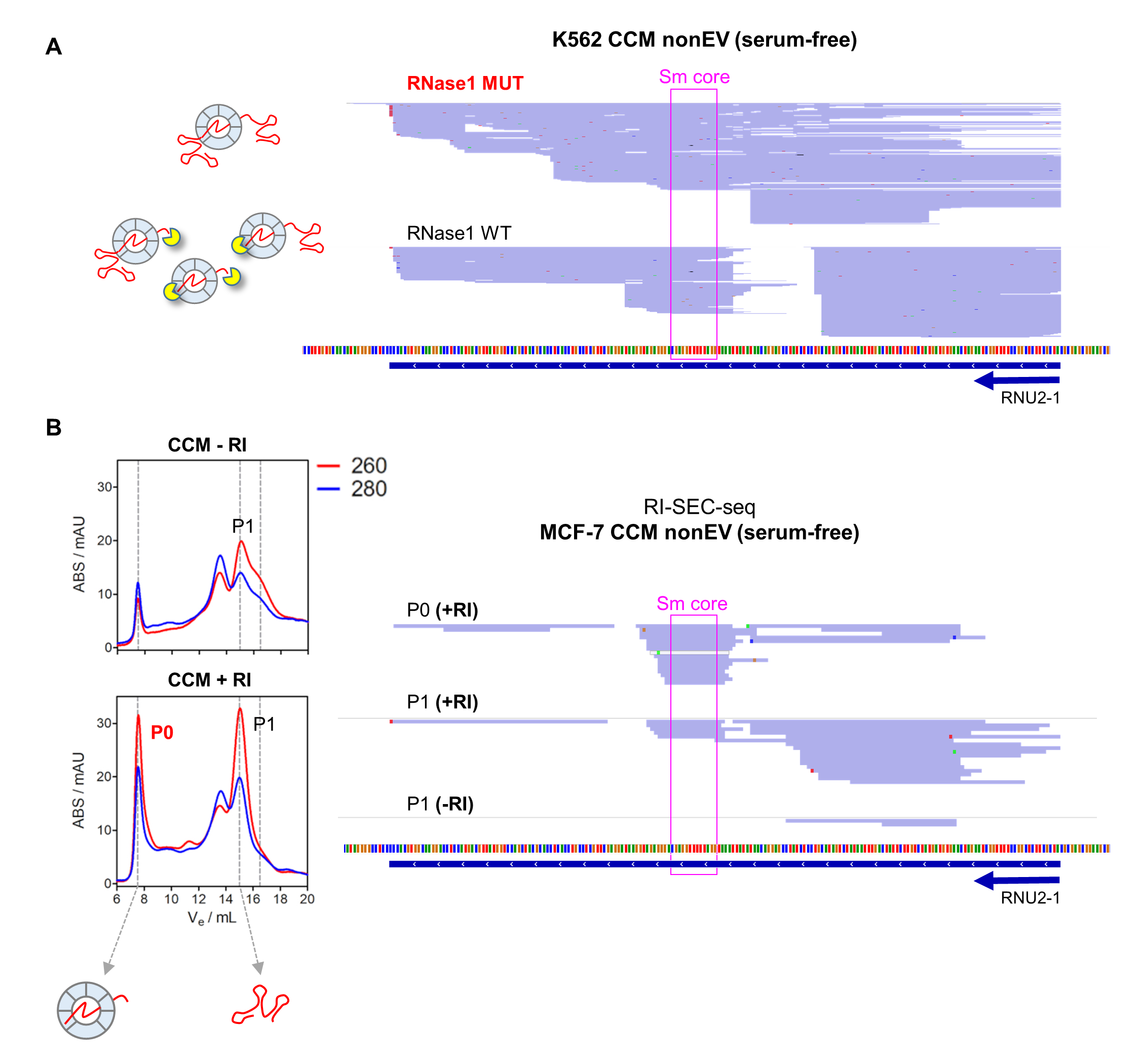

Supplement: Supplementary file 2 — Supplementary Figure 2: A) Sequence coverage plot of RNU2‐1 in nonvesicular extracellular samples (100,000 x g supernatants) of RNase 1‐null (top) and wild‐type (bottom) K562 cells grown under serum‐free conditions. Source data is from Nechooshtan et al. (2020) (SRA: SRR11539127 and SRR11539120, respectively). The diagram represents Sm proteins and the U2 snRNA (red), and the action of extracellular ribonucleases (yellow). B) Sequence coverage plot of RNU2‐1 in nonvesicular extracellular samples from MCF‐7 cells, further separated by size‐exclusion chromatography. Chromatograms are shown on the left. Serum‐free, vesicle‐depleted cell‐conditioned medium (CCM) was either treated or not with ribonuclease inhibitors (+/‐ RI). Selected peaks were then subjected to small RNA‐seq: “P0” (corresponding to the exclusion volume of a Superdex 200 column) and “P1”, corresponding to the elution volume of free tRNAs and other RNAs or RNA fragments of similar size. Source data is from Tosar et al. (2020). As in Figure 1, the blue‐coloured reads correspond to the minus DNA strand and their orientation (5′‐3′) is therefore right‐to‐left. The blue arrow indicates the start and direction of RNU2‐1 and the Sm core sequence is indicated in magenta. [file JEX2-1-e45-s004.tif]

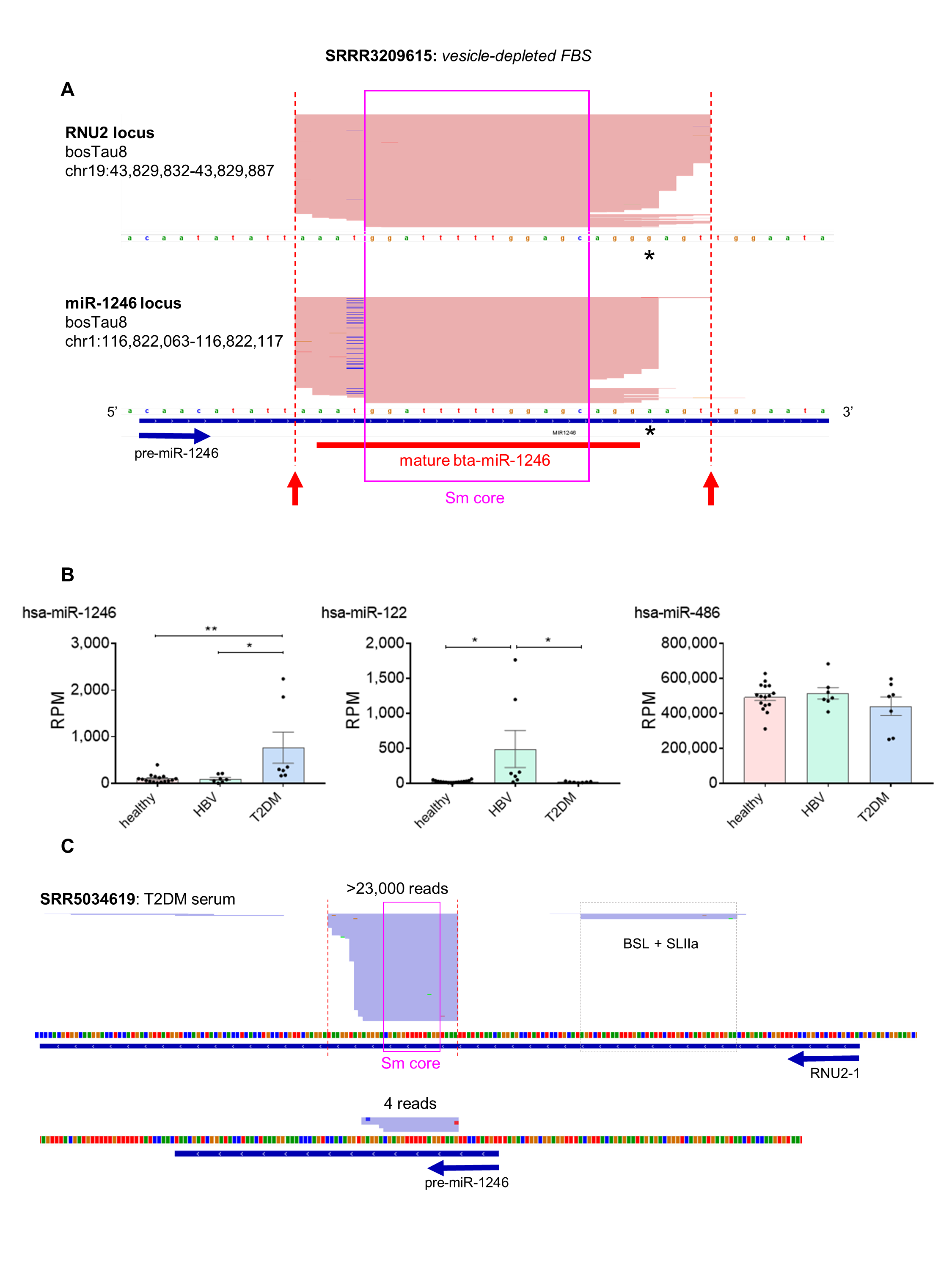

Supplement: Supplementary file 3 — Supplementary Figure 3: A) Sequence coverage plot of two loci in the bovine genome corresponding to RNU2 or a RNU2 pseudogene (top) and miR‐1246 (bottom). The blue arrow indicates the start and direction of the pre‐miR‐1246 sequence. Reads in the forward orientation (red) are from SRRR3209615 (100,000 x g supernatants of 10% FBS; Wei et al. 2016). Mature miR‐1246 was shown in Wei et al. 2016 as the second most abundant miRNAs in FBS. However, reads mapping to miR‐1246 are derived from at least two genomic loci (based on the presence of a mismatch, indicated by an asterisk) and do not correspond to the mature bta‐miR‐1246 sequence (red bar). The Sm core sequence is indicated in magenta and predicted cleavage sites 3′ to the most proximal pyrimidines outside of the Sm core are indicated with an arrow. B) LiqDB analysis of hsa‐miR‐1246 (left), hsa‐miR‐122 (centre) and hsa‐miR‐486 (right) in the sera of healthy donors or patients with Hepatitis B virus (HBV) or type 2 diabetes mellitus (T2DM). RPM: reads per million mapped reads. Source data is from Krauskopf et al. (2017). Hsa‐miR‐122 is included as a control because it is known to be affected in HBV‐infected patients. Hsa‐miR‐486 is a red blood cell‐derived miRNA that is abundant in serum and serves to show the overall number of miRNA‐mapping reads in the three categories are comparable. Thus, T2DM patients seem to have specifically high levels of hsa‐miR‐1246. However, liqDB is counting reads derived from the RNU2‐1 locus as miR‐1246. When analysing sequence coverage plots (C) of the RNU2‐1 and the miR‐1246 loci in a T2DM patients with unusually high levels of miR‐1246 (SRA: SRR5034619), most of the reads correspond to RNU2‐1. Again, most of these reads correspond to the Sm core region (magenta) flanked by additional, mostly purine residues (predicted cleavage sites 3′ to proximal pyrimidines are indicated). However, some reads corresponding to the branch site loop (BSL) and the stem loop IIa (SLIIa) are also present [file JEX2-1-e45-s001.tif]

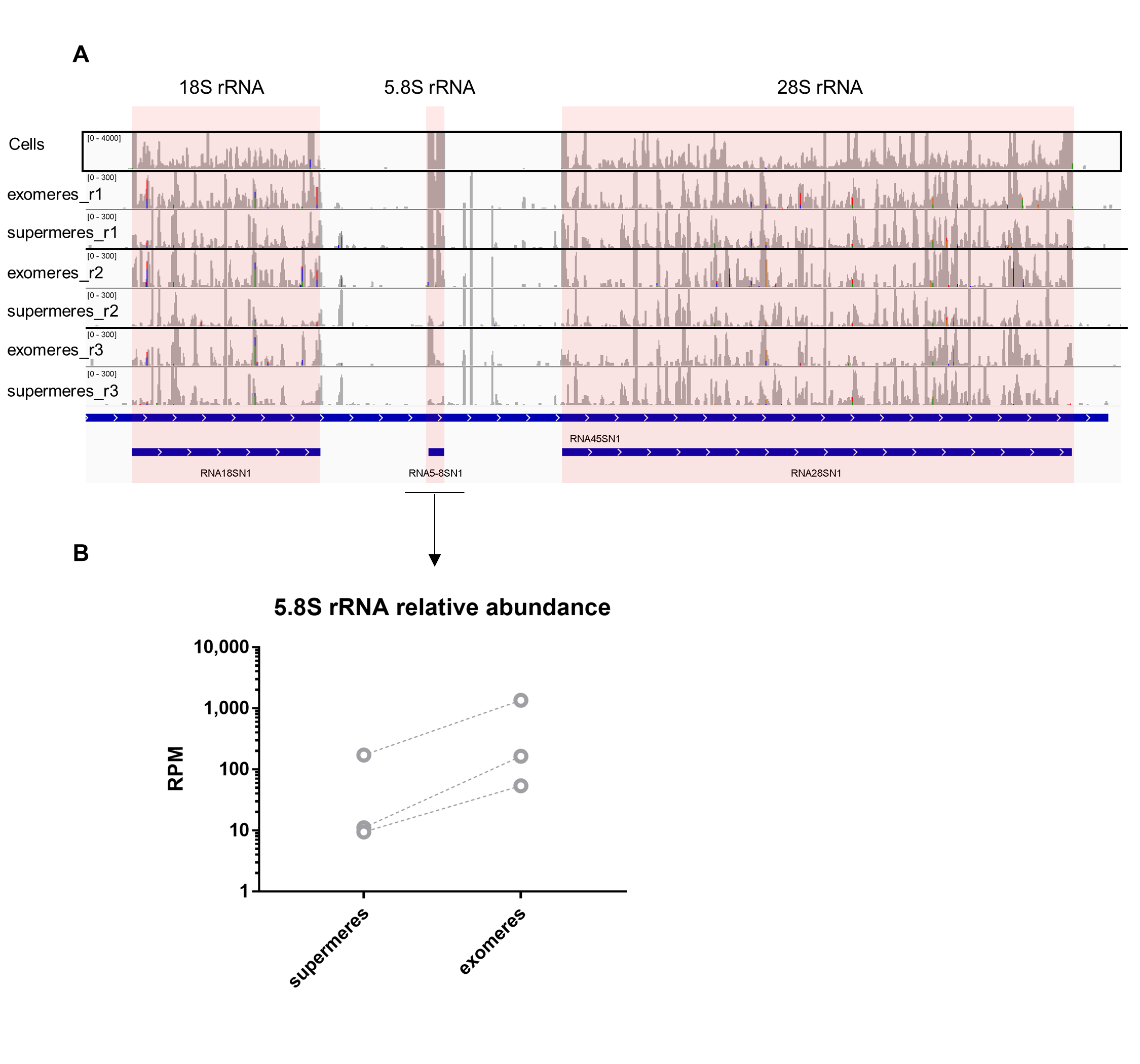

Supplement: Supplementary file 4 — Supplementary Figure 4: A) Sequence coverage plots of the 45S pre‐ribosomal RNA, containing the regions corresponding to the 18S, 5.8S and 28S rRNAs (from left to right in 5′‐3′ orientation). Source data is from Zhang et al. (2021). Unlike Figure 2D, the coverage plots were truncated at 300 reads (except in cells, where this number was set to 4000) in order to observe all the regions producing a relevant number of reads rather than those regions with the highest sequence coverage. B) Comparison of the total number of reads mapping to the 5.8 rRNA gene (in Log10 scale) in supermeres vs exomeres. Dashed lines connect paired samples (i.e., replicates 1, 2 and 3). [file JEX2-1-e45-s002.tif]
